# Comparative analysis of Fusarium crown rot resistance in synthetic hexaploid wheats and their parental genotypes

**DOI:** 10.1186/s12864-023-09268-7

**Published:** 2023-04-05

**Authors:** Ying Chen, Yunpeng Wang, Fangnian Guan, Li Long, Yuqi Wang, Hao Li, Mei Deng, Yazhou Zhang, Zhien Pu, Wei Li, Qiantao Jiang, Jirui Wang, Yuming Wei, Jian Ma, Qiang Xu, Houyang Kang, Pengfei Qi, Zhongwei Yuan, Lianquan Zhang, Dengcai Liu, Youliang Zheng, Guoyue Chen, Yunfeng Jiang

**Affiliations:** 1 State Key Laboratory of Crop Gene Exploitation and Utilization in Southwest China, Wenjiang, Chengdu, 611130 Sichuan P. R. China; 2 Triticeae Research Institute, Sichuan Agricultural University, Wenjiang, Chengdu, 611130 Sichuan P. R. China; 3 Dazhou Academy of Agricultural Sciences, Tongchuan, Dazhou, 635000 Sichuan P. R. China; 4 College of Agronomy, Sichuan Agricultural University, Wenjiang, Chengdu, 611130 Sichuan P. R. China

**Keywords:** Synthetic hexaploid wheat, Fusarium crown rot, Transcriptome, Phenylalanine ammonia-lyase

## Abstract

**Background:**

Fusarium crown rot (FCR) is a chronic disease of cereals worldwide. Compared with tetraploid wheat, hexaploid wheat is more resistant to FCR infection. The underlying reasons for the differences are still not clear. In this study, we compared FCR responses of 10 synthetic hexaploid wheats (SHWs) and their tetraploid and diploid parents. We then performed transcriptome analysis to uncover the molecular mechanism of FCR on these SHWs and their parents.

**Results:**

We observed higher levels of FCR resistance in the SHWs compared with their tetraploid parents. The transcriptome analysis suggested that multiple defense pathways responsive to FCR infection were upregulated in the SHWs. Notably, phenylalanine ammonia lyase (PAL) genes, involved in lignin and salicylic acid (SA) biosynthesis, exhibited a higher level of expression to FCR infection in the SHWs. Physiological and biochemical analysis validated that PAL activity and SA and lignin contents of the stem bases were higher in SHWs than in their tetraploid parents.

**Conclusion:**

Overall, these findings imply that improved FCR resistance in SHWs compared with their tetraploid parents is probably related to higher levels of response on PAL-mediated lignin and SA biosynthesis pathways.

**Supplementary Information:**

The online version contains supplementary material available at 10.1186/s12864-023-09268-7.

## Background

Hexaploid wheat, or common wheat (*Triticum aestivum*, AABBDD), is the youngest allohexaploid formed nearly 10,000 years ago as a hybrid between an early-cultivated tetraploid wheat (*Triticum turgidum*, BBAA) and diploid wild goatgrass (*Aegilops tauschii*, DD) [1, 2]. Compared with its tetraploid wheat progenitor, hexaploid wheat has greater physiological and ecological plasticity because of its higher ploidy level and complex genome composition [3]. Hexaploid wheat has a broader adaptability to different photoperiod and vernalization requirements than tetraploid wheat. It also has better stress tolerance to biotic and abiotic stresses [3]. Owing to this broader adaptability, hexaploid wheat has spread rapidly and is now one of the most important food crops worldwide [4, 5].

Fusarium crown rot (FCR), primarily caused by *Fusarium pseudograminearum*, is a soil-borne disease that seriously impacts wheat and barley production. This disease was first reported in Australia and now, it has become a prevalent disease, threating cereal production worldwide [6, 7]. Significant yield losses due to FCR have been documented globally. In Australia, estimated losses of wheat and barley due to FCR are approximately 97 million AUD, with total crop production losses of 10–20% reported [8, 9]. According to data from the Pacific Northwest in the United States, yield losses may be as high as 35% in winter wheat varieties under natural inoculum levels [10]. FCR has recently become one of the most important wheat diseases in the Huanghuai wheat-growing region since 2010 and affects different provinces with varying severity. For example, the highest frequency of FCR occurrence was observed in Henan province, where Xu et al. (2016) [11] reported that the diseased field ratio and white head ratio caused by FCR were 65.1% and 0.1%-31.5% in 2016, respectively. The yield loss in some affected fields reached as high as 51.6%. Although significant progress has been made on genetic resistance [12], currently, no wheat cultivars are completely immune to FCR.

A better understanding of the mechanisms of the host defenses against *F. pseudograminearum* can provide new strategies to develop varieties with better resistance to FCR. In wheat, several transcriptome analyses have been performed to investigate defense responses in wheat after FCR infection [13–15]. For example, comparative transcriptome analysis identified that genes encoding anti-microbial proteins, oxidative stress-related proteins, signaling molecules, and proteins involved in both primary and secondary metabolism were associated with FCR resistance [13]. In a combined transcriptome and metabolite analysis, Powell et al. (2017) found that genes associated with pathogen sensing and signaling, including transcription factor, cellular transport, and detoxification genes, were differentially expressed in the Australian bread wheat cultivar Chara after inoculation with FCR [14]. More recently, integrated transcriptome and metabolite analysis of Chinese wheat cultivars, highlighted that the benzoxazinoid biosynthesis pathway contributes to FCR resistance in wheat [15]. In fact, many responses observed in wheat during *F. pseudograminearum* infection are very similar to those induced by *F. graminearum* and *F. culmorum* [7].

An interesting phenomenon is that hexaploid wheat is generally more resistant to FCR infection than tetraploid wheat. Differences in FCR resistance between tetraploid and hexaploid wheat have been uncovered by several studies. For instance, Daniel et al. (2008) reported that FCR inoculation reduced the yield of durum wheat by 58%, whereas the average yield loss in bread wheat was only 25% across different soil types [16]. Liu et al. (2012) reported that durum wheat has a similar FCR severity as that of barley, whereas bread wheat exhibits less severe FCR stem-base browning symptoms [17]. Ma et al. (2012) assayed the FCR resistance of 2,500 wheat genotypes and found highly resistant genotypes in hexaploid wheat but not in tetraploid wheat [18]. The underlying reasons for the differences between tetraploid and hexaploid wheat are still not clear.

Recent studies have shown that reprogramming of transcriptomes, which occurs in newly formed hexaploid wheat, has distinct effects on growth vigor and growth adaptation following allohexaploidization [4, 19–21]. In this study, we compared the FCR responses of 10 synthetic hexaploid wheats (SHWs) (AABBDD) and their parental genotypes, *T. turgidum* (AABB) and *Ae. tauschii* (DD). We performed transcriptome analyses of two SHWs and their tetraploid and diploid parents following FCR infection to identify genes and pathways associated with improved FCR resistance of SHWs. We found evidence that defense pathways regulated by phenylalanine ammonia lyase (PAL) genes are altered in SHWs compared with their tetraploid and diploid parents. We also measured six physiological parameters related to FCR resistance. Our findings revealed the genetic basis of improved FCR resistance in SHWs, which may help to develop highly FCR-resistant varieties in future.

## Materials and methods

### Plant materials

In the study, 10 sets of SHWs and their tetraploid parents (including *T. turgidum* ssp. *dicoccoides*, *T. turgidum* ssp. *turgidum* and *T. turgidum* ssp. *dicoccon*) and diploid parents (including *Ae. tauschii* ssp. *tauschii* and *Ae. tauschii* ssp. *strangulata*) were used (Table 1). The tetraploid wheat *T. turgidum* (AABB) and diploid goat grass *Ae. tauschii* (DD) were used as parental lines to generate SHWs [22]. *T. turgidum* was used as a maternal parent and pollinated with *Ae.tauschii* anthers. S_1_ seeds were obtained from triploid F_1_ hybrids (ABD) after spontaneous chromosome doubling and were grown as the first generation of allohexaploid plants (AABBDD) [22]. Only plants with complete sets of chromosomes (28 from *T. turgidum* and 14 from *Ae. tauschii*) were used for further experimentation. Spontaneous chromosome doubling resulted from the union of un-reduced gametes [23]. No embryo rescue procedure or hormone treatment was applied for the production of the triploid F_1_ hybrid. All materials were preserved and provided by Triticeae Research Institute of Sichuan Agricultural University.

**Table 1:** FCR severity assessment of 10 SHWs and their tetraploid and diploid parents

Number of materials	Combination of materials	Ploidy level	Species	Disease index (%)	others
SHW-1	AS2308×AS72	hexaploid	Synthetic hexaploid wheat	38.5 ± 10.6	syn-SAU-83
TW-1	AS2308	tetraploid	*T. turgidum* ssp. *turgidum*	63.6 ± 15.0	Sichuan, China
DW-1	AS72	diploid	*Ae. tauschii* ssp. *tauschii*	51.4 ± 14.9	Xinjiang, China
SHW-2	AS286×AS2404	hexaploid	Synthetic hexaploid wheat	40.7 ± 5.0	syn-SAU-32
TW-2	AS286	tetraploid	*T. turgidum* ssp. *dicoccoides*	57.3 ± 4.6	France
DW-2	AS2404	diploid	*Ae. tauschii* ssp. *strangulata*	53.8 ± 16.0	TQ-29
SHW-3	AS2298×AS79	hexaploid	Synthetic hexaploid wheat	45.1 ± 10.5	syn-SAU-81
TW-3	AS2298	tetraploid	*T. turgidum* ssp. *turgidum*	74.1 ± 16.5	Sichuan, China
DW-3	AS79	diploid	*Ae. tauschii* ssp. *tauschii*	49.2 ± 11.6	Henan, China
SHW-4	AS2380×AS95	hexaploid	Synthetic hexaploid wheat	46.5 ± 15.3	syn-SAU-103
TW-4	AS2380	tetraploid	*T. turgidum* ssp. *turgidum*	65.3 ± 21.7	Shannxi, China
DW-4	AS95	diploid	*Ae. tauschii* ssp. *tauschii*	62.6 ± 7.0	AASC-13
SHW-5	AS2240×AS84	hexaploid	Synthetic hexaploid wheat	26.3 ± 2.5	syn-SAU-78
TW-5	AS2240	tetraploid	*T. turgidum* ssp. *turgidum*	49.0 ± 14.4	Sichuan, China
DW-5	AS84	diploid	*Ae. tauschii* ssp. *tauschii*	53.2 ± 6.4	AASC-2
SHW-6	AS2310×AS60	hexaploid	Synthetic hexaploid wheat	38.8 ± 7.5	syn-SAU-85
TW-6	AS2310	tetraploid	*T. turgidum* ssp. *turgidum*	55.4 ± 14.5	Sichuan, China
DW-6	AS60	diploid	*Ae. tauschii* ssp. *tauschii*	38.5 ± 14.4	Middle East
SHW-7	AS2295×AS76	hexaploid	Synthetic hexaploid wheat	42.0 ± 10.8	syn-SAU-79
TW-7	AS2295	tetraploid	*T. turgidum* ssp. *turgidum*	58.2 ± 14.8	Sichuan, China
DW-7	AS76	diploid	*Ae. tauschii* ssp. *tauschii*	59.0 ± 8.3	Shannxi, China
SHW-8	AS2255×AS93	hexaploid	Synthetic hexaploid wheat	45.5 ± 4.4	syn-SAU-12
TW-8	AS2255	tetraploid	*T. turgidum* ssp. *turgidum*	61.5 ± 6.6	China
DW-8	AS93	diploid	*Ae. tauschii* ssp. *tauschii*	54.6 ± 23.8	AASC-11
SHW-9	PI154582×AS95	hexaploid	Synthetic hexaploid wheat	50.0 ± 13.6	syn-SAU-95
TW-9	PI154582	tetraploid	*T. turgidum* ssp. *dicoccon*	70.5 ± 4.8	Taiwan
DW-9	AS95	diploid	*Ae. tauschii* ssp. *tauschii*	62.6 ± 7.0	AASC-13
SHW-10	AS2255×AS60	hexaploid	Synthetic hexaploid wheat	45.0 ± 12.7	SHW-L1
TW-10	AS2255	tetraploid	*T. turgidum* ssp. *turgidum*	61.5 ± 6.6	China
DW-10	AS60	diploid	*Ae. tauschii* ssp. *tauschii*	38.5 ± 14.4	Middle East

Note: “SHW” represents synthetic hexaploid wheat, “TW” represents tetraploid wheat, “DW” represents diploid wheat, “TW-8” is the same as “TW-10”, “DW-4” is the same as “DW-9”, “DW-6” is the same as “DW-10”.

### FCR inoculation and assessment

A *Fusarium pseudograminearum* isolate (NLYY), collected from Henan Province of China, was provided by Henan Institute of Science and Technology. The inoculum preparation, inoculation and FCR assessment were performed as described previously [24]. Briefy, inoculum was prepared on ½ strength potato dextrose agar. Inoculated plates were kept for 12 days at room temperature, and then mycelium was harvested. The mycelia were oscillated in Carboxymethylcellulose sodium Fluid Medium for 5–7 days before being filtered out with sterile gauze and spores were harvested. The concentration of spore suspension was adjusted to 1 × 10^6^ spores/mL and then used directly for inoculation or stored at − 20 °C until needed. Tween 20 was added (0.1% v/v) to the spore suspension prior to use.

Seedlings at 4 days post-germination, were inoculated with *Fusarium pseudograminearum* isolate (*Fp* inoculation) or distilled water (mock inoculation) following the protocol described previously [25]. Ten seedlings were used per biological replication with three replications. Briefly, Seeds were treated with 10% available hypochlorite solution for 20 min and then were thoroughly rinsed with distilled water. Surface sterilised seeds were then placed on three layers of filter paper saturated with water and left to germinate. Seedlings (4 days post-germination) were immersed in either spore suspension (*Fp* inoculation) or in distilled water (mock inoculation) for 1 min and seedlings were planted into the 50-well plastic trays containing sterilised nutrient soil. The plastic trays were arranged in the incubator and the settings for the incubator were: 25/16 (± 1) °C day/night temperature and 65%/85% day/night relative humidity.

FCR severity was assessed on a 0–5 scale at fourth weeks after inoculation, where “0” represents no symptom and “5” represents whole seedling completely necrotic [24]. A disease index (DI) was then calculated for each line following the formula: DI=(∑n_X_/5 N)×100, where X is the scale value of each plant, n is the number of plants in the category, and N is the total number of plants assessed for each material.

### RNA sequencing and expression analysis

Two SHWs (SHW-1 from the cross of *T. turgidum* ssp. *turgidum* and *Ae. tauschii* ssp. *tauschii*, and SHW-2 from the cross of *T. turgidum* ssp. *dicoccoides* and *Ae. tauschii* ssp. *strangulata*) and their tetraploid and diploid parents were selected for RNA-seq. The experimental design for each material contained two treatments (mock and *Fp* inoculation), one time point (3 days post inoculation) and three biological replicates. And a total of ten samples for each biological replicate were used for RNA extraction. Samples were harvested by cutting the stem bases (1 cm) at 3 days post inoculation and snap-frozen in liquid nitrogen and kept at − 80 °C until processed. Total RNA was isolated using a HiPure HP Plant RNA Mini Kit (Magen, R4165–02) according to the manufacturer’s instructions.

A total of 36 libraries were sequenced in the 150 bp paired-end mode using Illumina NovaSeq6000 platform at Berry Genomics Corporation (Beijing, China). Sequence files were deposited at the National Genomics Data Center (NGDC) Genome Sequence Archive under BioProject ID PRJCA011781 (https://ngdc.cncb.ac.cn/search/). Sequence quality assessment, trimming and RNA-seq analysis was performed using CLC Genomics 12.0 software with default parameters. The processed RNA-seq data in SHWs were aligned to complete mRNA (cDNA) sequences of Chinese Spring Reference genome IWGSCv1.1 (a total of 105,200 genes). While RNA-seq data in tetraploid parents and diploid parents were respectively aligned to mRNA sequences of AABB sub-genomes (70,988 genes) and DD sub-genome (34,212 genes) from Chinese Spring Reference genome IWGSCv1.1. The expression level of each gene was calculated using TPM (Transcripts Per Kilobase of exon model per Million mapped reads ). Differentially Expressed Genes (DEGs) were restricted with the absolute value of log_2_ (fold Change) ≥ 1 and False Discovery Rate (FDR) < 0.05 as the threshold by performing pairwise comparisons of *Fp* treated and corresponding mock samples. DEGs were separated into up-regulated and down-regulated genes and used as individual test sets for GO enrichment analysis. GO term enrichment analysis was done using Triticeae-GeneTribe (http://wheat.cau.edu.cn/TGT/) [26].

### RT-qPCR analysis

Thirteen DEGs were randomly selected and assessed using real-time quantitative PCR (RT-qPCR). Primers were designed using Primer-BLAST tool (http://www.ncbi.nlm.nih.gov/tools/primer-blast/) and listed in Table S1 (Additional file 1). EF-1α, elongation factor of wheat, was used as the internal reference gene. RNA isolation, cDNA synthesis and RT-qPCR analysis were performed as described in Wang et al. (2010) [27]. The relative fold changes were calculated using the comparative CT method (2^−∆∆CT^). Genes with Ct values > 40 cycles were regarded as having no expression value.

### Physiological and biochemical assessment

PAL activity, chitinase activity, salicylic acid (SA) content, jasmonic acid (JA) content and abscisic acid (ABA) content of 5 SHWs (SHW-1, SHW-2, SHW-3, SHW-4 and SHW-5) and their tetraploid and diploid parents were determined using the ELISA method at FANKEL Industrial Corporation (Shanghai, China). The experimental design for each line contained one treatment (*Fp* inoculation), one time point (3 days post inoculation) and three biological replicates. And a total of ten samples for each biological replicate were used. Samples were harvested by cutting the stem bases and snap-frozen in liquid nitrogen and kept at − 80 °C until processed.

Lignin content of 10 SHWs and their tetraploid parents was measured by ultraviolet spectrophotometry at FANKEL Industrial Corporation (Shanghai, China). To measure basal lignin levels in stem bases, the experimental design for each line contained two time points (seedling and heading stage) and three biological replicates. And a total of ten samples for each biological replicate were used. Samples were harvested by cutting the stem bases and dried to constant weight.

### Statistical analysis

Paired samples T-test was performed in Excel 2019 and significant differences was claimed at *p* < 0.05.

## Results

### Assessment of FCR severity in SHWs and their parents

**Fig. 1:**
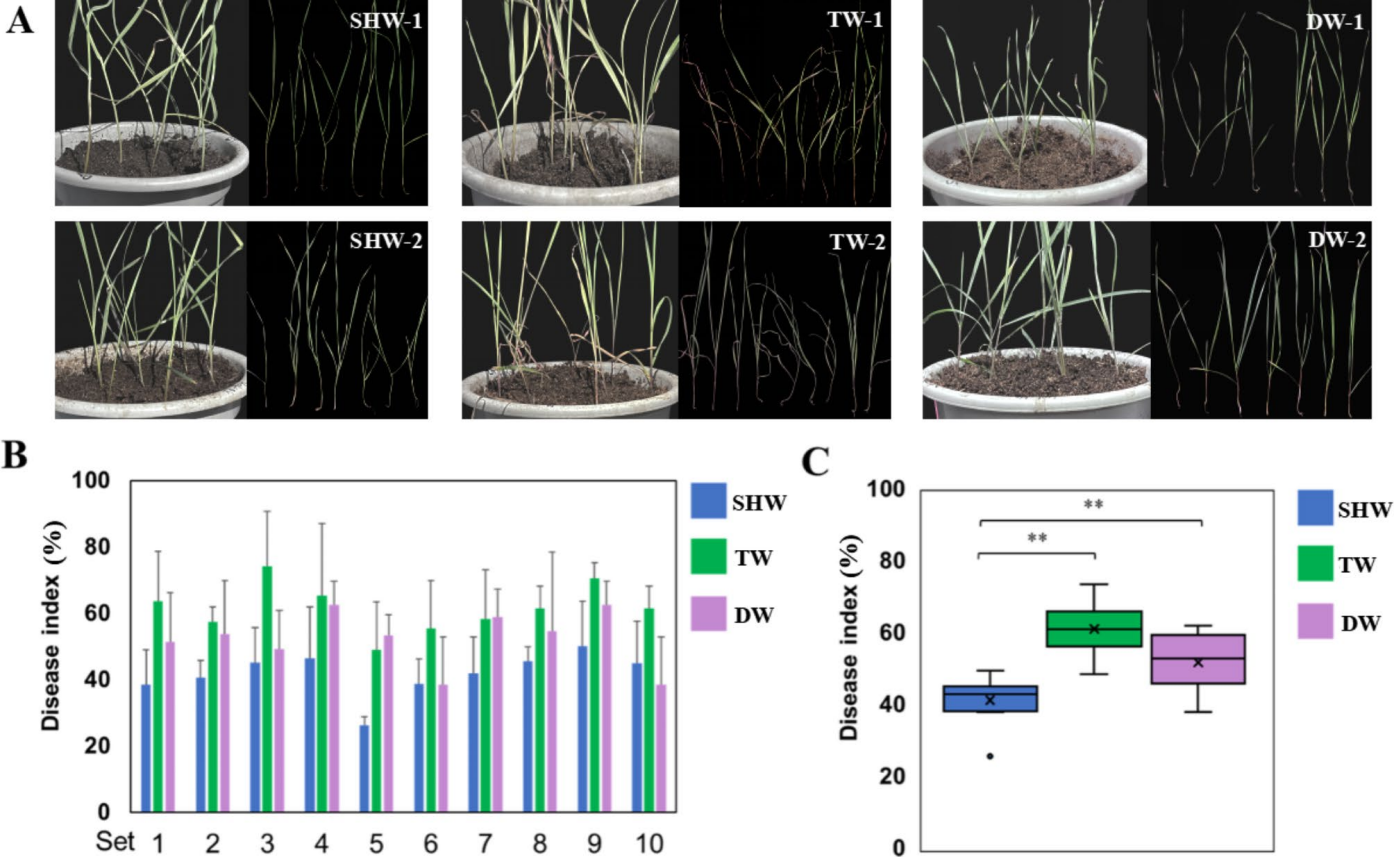
**– The difference in FCR severity among SHWs and their tetraploid and diploid parents.** (A) A representative figure of the FCR responses in 2 SHWs and their tetraploid and diploid parents. (B) DI values of 10 SHWs and their tetraploid and diploid parents. (C) Box plot displays average DI values of 10 SHWs and their tetraploid and diploid parents. **p < 0.01. “SHW” represents synthetic hexaploid wheat, “TW” represents tetraploid wheat, “DW” represents diploid wheat

In this study, we performed an assessment of the FCR severity of 10 SHWs and their parents at the seedling stage in a greenhouse. Eight SHWs had lower disease index (DI) values than their diploid parents, whereas all SHWs had lower DI values than their tetraploid parents (Fig. 1A–B). Average DI values of SHWs, tetraploid parents, and diploid parents were 41.8 (26.3–50.0), 61.6 (49.0–74.1), and 52.3 (38.5–62.6), respectively (Table 1; Fig. 1B–C). Compared with those of their tetraploid parents, DI values of SHWs were reduced by 16.0–29.0%. Clearly, SHWs had significant higher levels of resistance to FCR (*p* < 0.01) infection than their parents (Fig. 1C).

### Transcriptomic response of two SHWs and their parents to FCR infection

To gain insight of the molecular mechanism of the improved FCR resistance in hexaploid wheat, we performed RNA-seq analysis on two SHWs and their corresponding tetraploid and diploid donors. An average of 12.9, 13.1, and 12.3 Gb of sequencing data were generated from the SHWs, tetraploid parents, and diploid parents, respectively (Additional file 1: Table S2). The filtered clean reads were mapped to the Chinese Spring reference genome (IWGSC 1.1). Average mapped ratios of SHWs and their tetraploid and diploid parents were 98.6%, 87.2%, and 87.4%, respectively (Additional file 1: Table S2). Most reads from all samples were successfully mapped, thus indicating that the selected reference genome (IWGSC 1.1) sufficiently reflected the genomic expression of the SHWs and their parents.

To verify the RNA-seq results, we carried out a RT-qPCR analysis using 13 genes (10 up-regulated and 3 down-regulated) randomly selected from the DEGs of two SHWs (Additional file 1: Table S1). Results of RT-PCR analysis were in agreement with the RNA-seq analysis (Additional file 2: Figure S1).

**Fig. 2:**
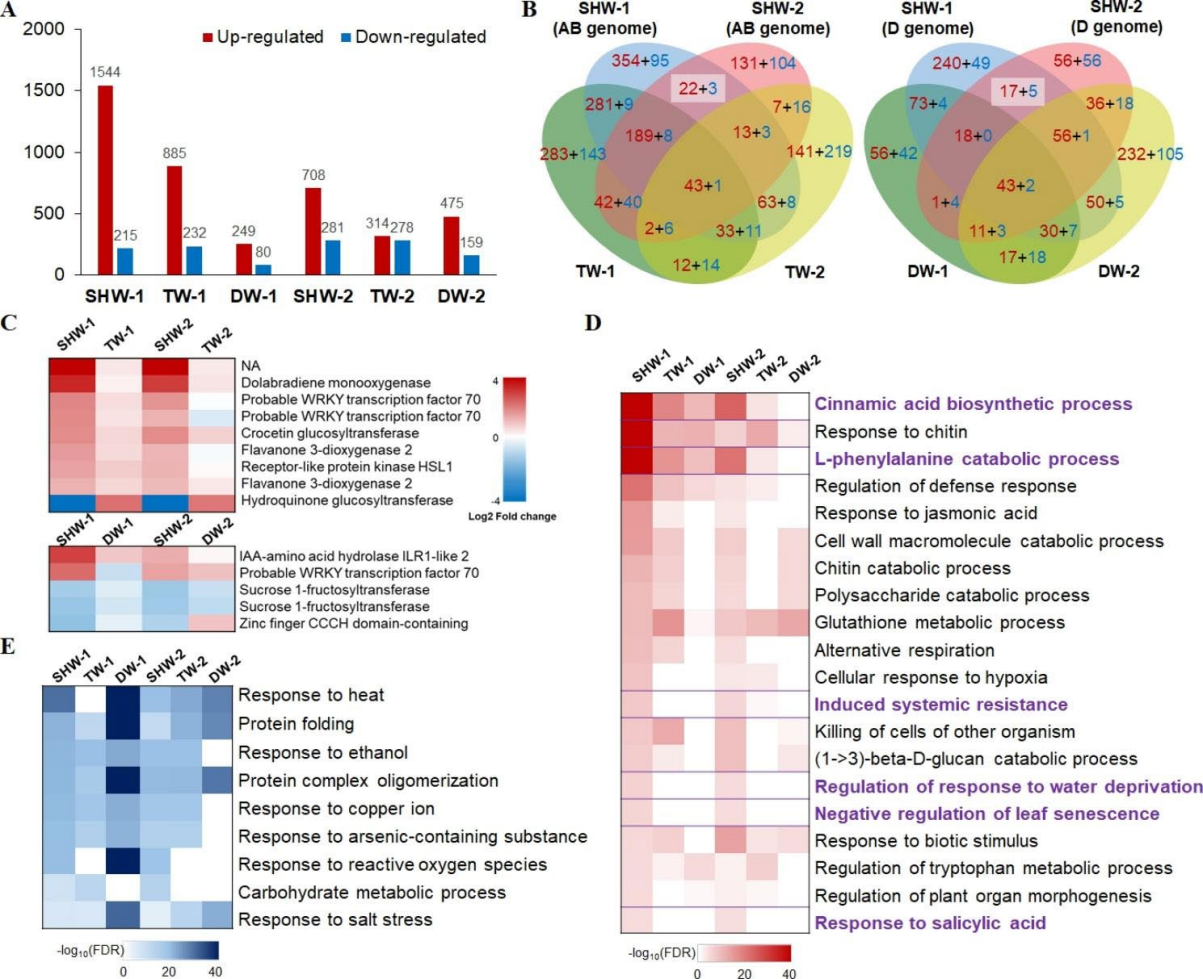
**– Transcriptome differences among 2 SHWs and their tetraploid and diploid parents after FCR infection.** (A) The number of up-regulated and down-regulated differentially expressed genes (DEGs). (B) Venn diagrams showing the number of DEGs among 2 SHWs and their tetraploid and diploid parents. Red and blue indicated the number of up-regulated and down-regulated DEGs, respectively. (C) Heat maps showing the identical and specific DEGs in SHWs. Log_2_ (fold change) in two SHWs ≥ 1 and all parents < 1, or two SHWs ≤ -1 and all parents > -1. (D) Heat maps showing the identical up-regulated GO terms in SHWs. Purple highlighted the differential GO terms among 2 SHWs and their parents. (E) Heat maps showing the identical down-regulated GO terms in SHWs.

The number of genes differentially expressed in response to FCR were much higher in SHWs than in their tetraploid and diploid parents, and most of these DEGs were up-regulated (Fig. 2A, Additional file 1: Table S3). A total of 431 DEGs (408 up-regulated and 23 down-regulated) were shared between the two SHWs, whereas 122 (90 up-regulated and 32 down-regulated) and 131 (101 up-regulated and 30 down-regulated) were identical between the tetraploid and diploid parents, respectively (Fig. 2B). These shared DEGs may be important signatures of basal resistance of wheat lines with different ploidy levels. In particular, 25 identical DEGs (22 up-regulated and 3 down-regulated) encoding proteins such as dolabradiene monooxygenase, WRKY transcription factors, flavanone 3-dioxygenase, receptor-like protein kinase HSL, and crocetin glucosyltransferase were highly detected in the AB subgenomes of the two SHWs (Fig. 2B–C). 22 identical DEGs from the D subgenome (17 up-regulated and 5 down-regulated) were highly detected in the two SHWs (Fig. 2B-C); these genes encode proteins such as IAA-amino acid hydrolase ILR1-like protein, WRKY transcription factors, sucrose 1-fructosyltransferase, and zinc finger CCCH domain-containing protein.

Gene ontology (GO) enrichment analysis was performed to classify the functions of DEGs. This analysis revealed that 90, 48, and 26 terms related to biological processes were enriched (FDR < 0.05) in up-regulated DEGs of SHW-1 and its tetraploid and diploid parents, respectively (Additional file 1: Table S4), whereas 24, 36, and 17 functional biological process categories were enriched in down-regulated DEGs (Additional file 1: Table S5). In regard to SHW-2 and its tetraploid and diploid parents, 62, 45, and 20 functional categories under biological process were respectively enriched in up-regulated DEGs (Additional file 1: Table S4), whereas 33, 31, and 14 categories were enriched in down-regulated DEGs (Additional file 1: Table S5). GO terms enriched in up-regulated DEGs of SHWs were more abundant than those of their parents. Several defense-related pathways were commonly enriched in up-regulated DEGs of SHWs and their tetraploid and diploid parents, including cinnamic acid biosynthetic process (GO:0009800), chitin catabolic process (GO:0006032), L-phenylalanine catabolic process (GO:0006559), regulation of defense response (GO:0031347), glutathione metabolic process (GO:0006749), response to oxidative stress (GO:0006979) (Fig. 2D), while pathways related to response to heat (GO:0009408), protein folding (GO:0006457), response to ethanol (GO:0045471), protein complex oligomerization (GO:0051259), and response to salt stress (GO:0009651) were enriched in down-regulated DEGs (Fig. 2E). Some pathways were significantly enriched only in up-regulated DEGs of SHWs, namely, induced systemic resistance (GO:0009864), regulation of response to water deprivation (GO:2,000,070), negative regulation of leaf senescence (GO:1,900,056), and response to salicylic acid (GO:0009751) (Fig. 2D). In addition, the categories of L-phenylalanine catabolic process (GO:0006559) and cinnamic acid biosynthetic process (GO:0009800) were more significantly enriched in DEGs of SHWs than in those of their tetraploid and diploid parents (Fig. 2D).

### Differences in expression patterns of defense pathways regulated by PAL genes among SHWs and their tetraploid and diploid parents

**Fig. 3:**
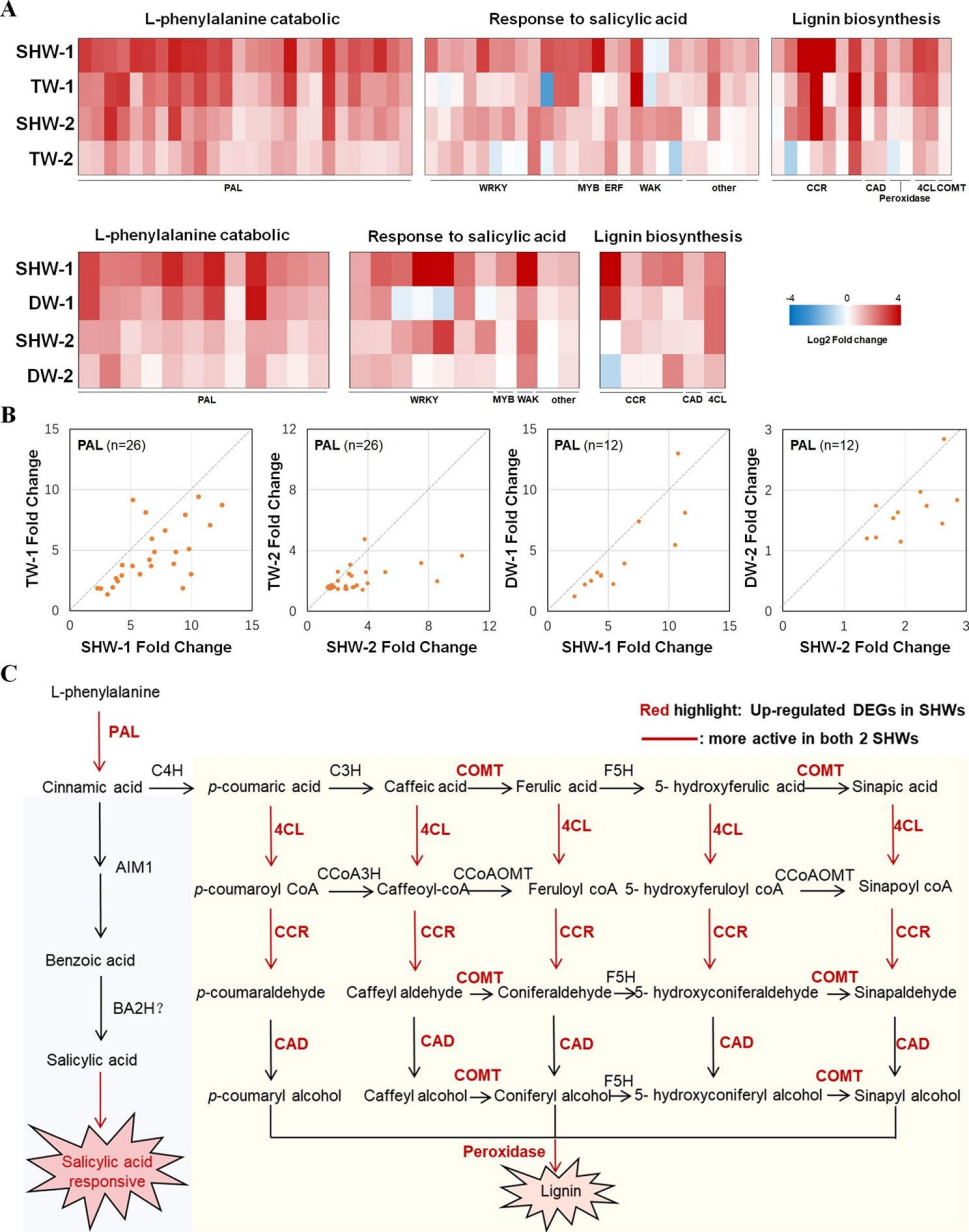
**– The difference of expression pattern in defense pathways regulated by Phenylalanine ammonia-lyase genes (PAL) among SHWs and their tetraploid and diploid parents.** (A) Expression of genes involved in L-phenylalanine catabolic process (GO:0006559), response to salicylic acid (GO:0009751) and lignin biosynthesis (GO:0009809) in two SHWs and their tetraploid and diploid parents following FCR infection. (B) Scatter plots of fold changes for PAL genes. n represents number of genes. (C) The difference of expression pattern in lignin and salicylic acid biosynthesis pathways between SHWs and their tetraploid parents. C4H, cinnamic acid 4-hydroxylase; C3H, coumarate 3-hydroxylase; 4CL, 4-coumarate coenzyme A ligase; CCoAOMT, caffeoyl CoA O-methyltransferase; CCoA3H: *p*-coumarate3-hydroxylase; CCR, cinnamoyl-CoA reductase; F5H, ferulate 5-hydroxylase; COMT, caffeic acid O-methyltransferase; CAD, cinnamyl alcohol dehydrogenase; AIM1, abnormal inflorescence meristem 1;BA2H, benzoic acid 2-hydroxylase

PAL, which plays an important role in lignin and salicylic acid biosynthesis, catalyzes the deamination of phenylalanine to form trans-cinnamic acid [28, 29]. L-phenylalanine catabolic process (GO:0006559) and cinnamic acid biosynthetic process (GO:0009800) are synonymous GO terms for functions that are mainly regulated by PAL genes. In this study, 38 PAL genes were found to be up-regulated DEGs in two SHWs (SHW-1 and SHW-2) following FCR infection; among these genes, 19 were significantly up-regulated in both SHWs. Almost all of these PAL genes had a stronger response to FCR infection in the SHWs than in their tetraploid and diploid parents (Fig. 3A–B). At the same time, many genes involved in SA response, such as those encoding WRKY transcription factors, MYB transcription factors, ethylene-responsive transcription factors, wall-associated receptor kinase, and NDR1/HIN1-like protein, had higher expression levels in the two SHWs than in their tetraploid and diploid parents following FCR infection (Fig. 3A). Several genes involved in the lignin biosynthesis pathway were also up-regulated in SHWs and their tetraploid and diploid parents following FCR infection (Fig. 3A). Specifically, part of genes involved in the lignin biosynthesis pathway were likely to be more active in both two SHWs following FCR infection (Fig. 3A, C), including genes encoding cinnamoyl-CoA reductase (TraesCS5A02G061600, TraesCS5A02G061700, TraesCS5B02G069600, TraesCS5B02G160700, TraesCS5D02G073300, TraesCS5D02G168400 and TraesCS7D02G150500), probable 4-coumarate-CoA ligase (TraesCS2A02G145800 and TraesCS2B02G171200), and peroxidase (TraesCS3A02G180000 and TraesCS7A02G353400).

### Physiological and biochemical parameter associated with resistance to FCR infection in SHWs and their parents

**Fig. 4:**
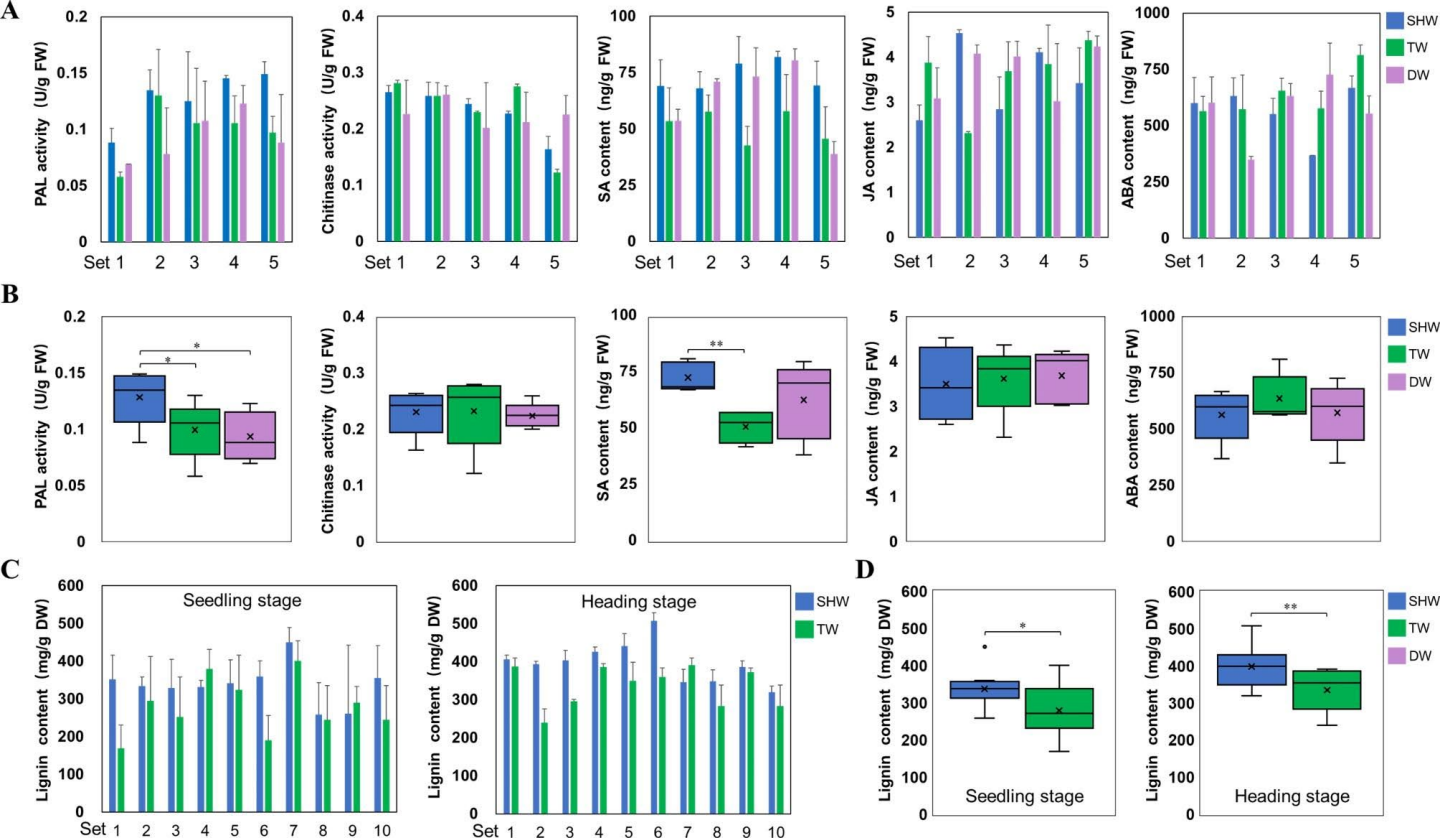
**– Measurements of six physiological parameters associated with resistance to FCR infection in SHWs and their parents.** (A) The bar graphs display PAL activity, chitinase activity, SA content, JA content and ABA content of the stem base in 5 SHWs and their parents following FCR infection, respectively. (B) The box plots display average PAL activity, chitinase activity, SA content, JA content and ABA content of the stem base in 5 SHWs and their parents following FCR infection, respectively. (C) The bar graphs display lignin content of the stem base in 10 SHWs and their tetraploid parents at seedling and heading stages, respectively. (D) The box plots display average lignin content of the stem base in 10 SHWs and their tetraploid parents at seedling and heading stage. *p < 0.05, **p < 0.01. “SHW” represents synthetic hexaploid wheat, “TW” represents tetraploid wheat, “DW” represents diploid wheat

Six physiological parameters related to FCR resistance were also analyzed in the SHWs and their parents. First, we measured PAL and chitinase activities and SA, JA, and ABA contents of the stem base of five SHWs and their parents following FCR infection. As expected, average PAL activities (0.1285 U/g FW) were significantly higher in SHWs than in their tetraploid parents (0.0992 U/g FW) and diploid parents (0.0933 U/g FW) (Fig. 4A–B, Additional file 1: Table S6). Moreover, all five SHWs had higher SA contents than observed in their tetraploid and diploid parents; in particular, average SA contents of the five SHWs, their tetraploid parents, and diploid parents were 73.37, 51.38, and 63.39 ng/g FW, respectively. In contrast to the significantly higher (*p* < 0.01) SA contents of SHWs compared with their tetraploid parents (Fig. 4A–B, Additional file 1: Table S6), no significant differences in chitinase activity, JA content, and ABA content were found among SHWs and their tetraploid and diploid parents (Fig. 4A-B, Additional file 1: Table S6). We also measured the total lignin content of the stem base at seedling and heading stages. Most of the 10 SHWs had higher lignin contents than those of their tetraploid parents at seedling or heading stages (Fig. 4C, Additional file 1: Table S7), and the overall average lignin content of the 10 SHWs was significantly higher than that of their tetraploid parents both at seedling (*p* < 0.05) and heading (*p* < 0.01) stages (Fig. 4D, Additional file 1: Table S7).

## Discussion

FCR has dramatically increased worldwide as a result of drier seasonal conditions and the adoption of moisture-preserving cultural practices, such as minimum tillage and stubble retention [6, 7]. Tetraploid wheat is especially susceptible to FCR, with few highly resistant varieties and germplasms currently available for production and breeding. Similar to salt [4] and N-deficiency [5] tolerances, the FCR resistance of hexaploid wheat is stronger than that of tetraploid wheat [16–18]. Nevertheless, little is known about the contributions of polyploidization to FCR resistance in wheat. In this study, we confirmed that improved FCR resistance in the SHWs compared with their tetraploid and diploid parents. This resistance has seen consistently in all 8 out of 10 SHWs (except SHW-6 and SHW-10) derived from different tetraploid and diploid parents (including three *T. turgidum* and two *Ae. tauschii* subspecies), thereby exhibiting transgressive performance over both diploid and tetraploid parents.

Transcriptome reprogramming due to the introduction of the D genome occurred in SHWs, leading in turn to global changes in gene expression [19]. These gene expression changes may have led to phenotypic differences between SHW and its parental species and may thus have been important sources of the dominant phenotypes of SHW, such as its growth vigor and adaptation [19, 20]. In this study, we performed transcriptome analyses of two SHWs and their tetraploid and diploid parents following FCR infection. We found that more DEGs and up-regulated GO terms in the SHWs than in their tetraploid and diploid parents following FCR infection. In particular, induced systemic resistance and response to salicylic acid were only significantly enriched in up-regulated DEGs of SHWs. This suggests that SHWs are more likely to have a stronger induced systemic resistance to FCR compared to their parents. Additionally, the L-phenylalanine catabolic process and cinnamic acid biosynthetic process were the most significantly enriched plant defense pathways against FCR infection, and they were commonly enriched in SHWs and their tetraploid and diploid parents. However, these pathways were likely more active in the SHWs than in their parents.

As an entry-point enzyme in the phenylpropanoid biosynthesis pathway, PAL converts phenylalanine to trans-cinnamic acid and free ammonia, thereby playing an important role in lignin and salicylic acid biosynthesis. Many studies have shown that PAL acts as a positive regulator of resistance to various plant biotic stresses [30–36]. In the transcriptome analyses in the present study, cinnamic acid biosynthesis (GO:0009800) and L-phenylalanine catabolic (GO:0006559) processes were significantly enriched both in SHWs and their parents following FCR infection. A total of 38 PAL genes were detected as up-regulated DEGs in two SHWs following FCR infection, and most of these PAL genes had higher levels of expression in the SHWs than in their tetraploid and diploid parents. Physiological measurements later confirmed that PAL activity was significantly higher in the SHWs than in their tetraploid and diploid parents following FCR infection, which suggests that the SHWs possess a stronger ability to induce PAL activity against FCR infection than their parents.

Lignin, an important component of the plant cell wall, provides an effective barrier for plants to resist pathogen infections and plays a crucial role in plant disease resistance [37]. A recent study has documented that loss of function of the dirigent gene *TaDIR-B1* improves resistance to FCR in wheat by increasing the accumulation of lignin [38], thus indicating that lignin plays an important role in FCR resistance. In our study, most up-regulated cinnamoyl-CoA reductase genes had stronger responses in SHWs than in their tetraploid and diploid parents following FCR infection, whereas higher lignin contents were found in the stem bases of the SHWs, both at seedling and heading stages. Alteration of PAL activity in SHWs could lead to changes in lignin contents and thicknesses of cell walls, making SHWs more resistant to FCR infection compared with their tetraploid parents.

The importance of SA in plant defense against pathogen attack has been thoroughly documented [35, 39–42]. Compared with that in tetraploid parents, higher PAL activity in SHWs is likely to increase levels of SA accumulation and SA-responsive gene expression following FCR infection. As expected, response to SA (GO:0009751) and induced systemic resistance (GO:0009864) were only significantly enriched in up-regulated DEGs of SHWs. Moreover, SA contents of the stem bases of the SHWs were significantly higher than those of their tetraploid parents following FCR infection, while there was no significant difference from their diploid parents. In addition, the SA content of diploid parents was intermediate between that of SHWs and tetraploid parents, consistent with the performance seen in our FCR severity assessment.

**Fig. 5:**
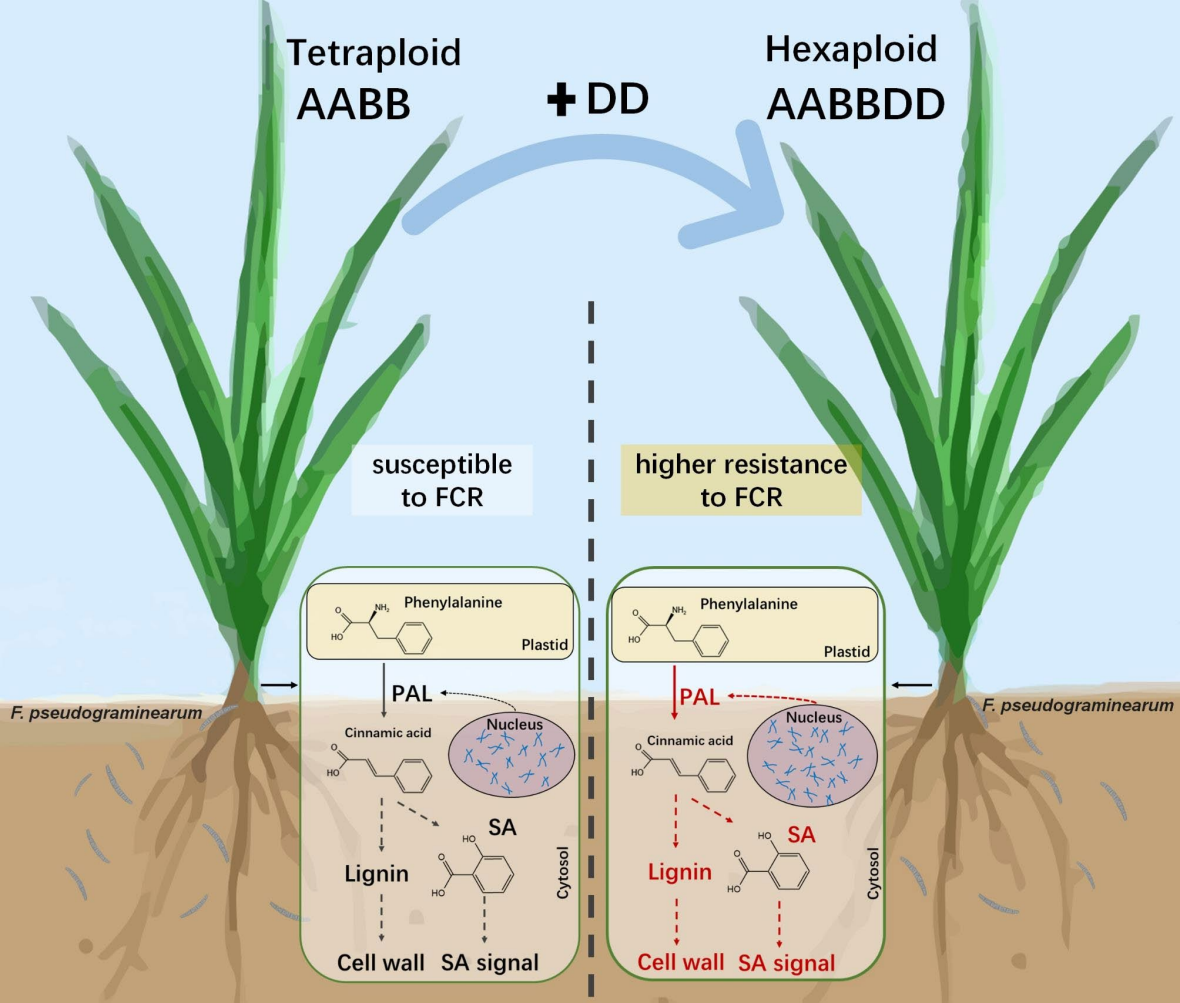
** A potential mechanism for improving Fusarium crown rot resistance in hexaploid wheat**. Red indicated stronger response pathways in hexaploid wheat than in tetraploid wheat following *F. pesudagraminearum* infection

Higher levels of response on PAL-mediated lignin and SA biosynthesis pathways may contribute to improved FCR resistance in SHWs compared with their tetraploid parents (Fig. 5). However, the altered defense pathways in SHWs may be diverse and include broader basal resistance. The changes of defense pathways in SHWs may involve two main reasons. One is the resistance effect of D genome genes from diploid parents. Our study has shown that diploid parents were more resistant and had higher SA content than tetraploid parents, indicating that stronger induced systemic resistance in SHWs may be mainly due to enhanced functionality of the D genome. This is consistent with a similar finding in SHWs under salt stress [4]. The other reason is an additive effect of genes from two parents. In fact, most genes showed additive expression in SHWs [43], providing an additive performance for many morphophysiological traits in SHWs. Similarly, SHWs exhibited stronger PAL activity than their tetraploid and diploid parents after FCR infection, which may be due to the presence of more PAL genes and their higher levels of expression in SHWs. Anyway, improved FCR resistance in SHWs which may be accomplished via multiple mechanisms that need further investigation.

Previous transcriptome analyses have revealed various genes and pathways associated with FCR resistance in wheat, such as anti-microbial proteins, oxidative stress-related proteins, signaling molecules, primary and secondary metabolism, pathogen sensing and signaling, cellular transport and detoxification, and benzoxazinoid biosynthesis [13–15]. However, these studies mainly focused on comparing different wheat genotypes or cultivars with different FCR resistance levels. Unlike previous findings, our study highlighted the advantage of hexaploid wheat on FCR resistance. The differential defense pathways identified between hexaploid and tetraploid wheat not only help elucidate the genetic basis of FCR resistance in hexaploid wheat but also shed light on the reasons for higher susceptibility in tetraploid wheat. These findings have potential implications for breeding improvement for FCR resistance, especially for highly susceptible tetraploid wheat.

## Conclusion

In this study, we compared the FCR responses of 10 SHWs and their parental genotypes, *T. turgidum* and *Ae. tauschii*. We confirmed improved FCR resistance in the SHWs compared with their parental genotypes. The transcriptome analysis of two SHWs and their tetraploid and diploid parents suggested that multiple defense pathways responsive to FCR infection were stronger in the SHWs than in the parents. Notably, dozens of PAL genes, involved in lignin and SA biosynthesis, exhibited a higher level of expression to FCR infection in the SHWs. Physiological and biochemical analysis validated that PAL activity and SA and lignin contents of the stem bases were higher in SHWs than their tetraploid parents. In summary, this study suggested that improved FCR resistance in SHWs compared with their tetraploid parents is probably related to higher levels of response on PAL-mediated lignin and SA biosynthesis pathways.

## Electronic supplementary material

Below is the link to the electronic supplementary material.

Additional file 1: **Table S1**. Primers for real-time quantitative PCR.
**Table S2**. RNA-seq read information and mapping statistics of 2 SHWs and their tetraploid and diploid parents.
**Table S3**. Differentially expressed gene (DEGs) of 2 SHWs and their tetraploid and diploid parents.
**Table S4**. GO terms enriched by up-regulated DEGs of 2 SHWs and their tetraploid and diploid parents.
**Table S5**. GO terms enriched by down-regulated DEGs of 2 SHWs and their tetraploid and diploid parents.
**Table S6**. PAL activity, chitinase activity, SA content, JA content and ABA content of the stem base in 5 SHWs and their parents following FCR infection.
**Table S7**. Total lignin content of the stem base of 10 SHWs and their tetraploid parents at the seedling and heading stage.

Additional file 2: **Figure S1**. Validation of data of RNA-seq by RT-qPCR

## Data Availability

Sequence files were deposited at the National Genomics Data Center (NGDC) Genome Sequence Archive under BioProject ID PRJCA011781.
