# Supplementary figures and images for: Comparative analysis of Fusarium crown rot resistance in synthetic hexaploid wheats and their parental genotypes
Source: BMC Genomics. 2023 Apr 5;24:178. doi: 10.1186/s12864-023-09268-7 (PMC10077658; doi:10.1186/s12864-023-09268-7)

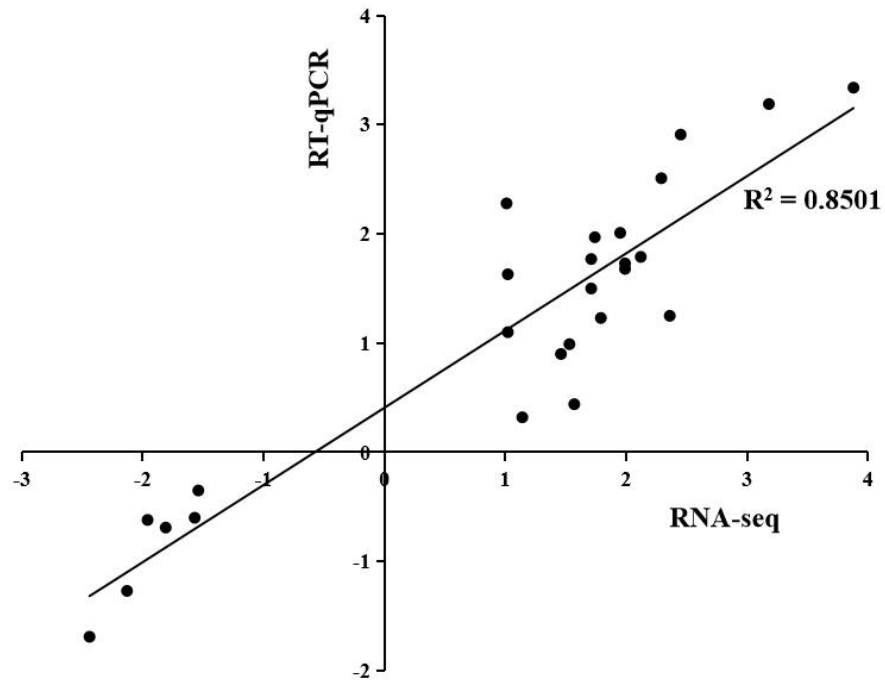

**Figure. S1 –Validation of data of RNA-seq by RT-qPCR.** The value represents the  $\log_2$  (fold change) of DEGs.

Supplement: Supplementary file 2 — Additional file 2: Figure S1. Validation of data of RNA-seq by RT-qPCR [file 12864_2023_9268_MOESM2_ESM.pdf]
